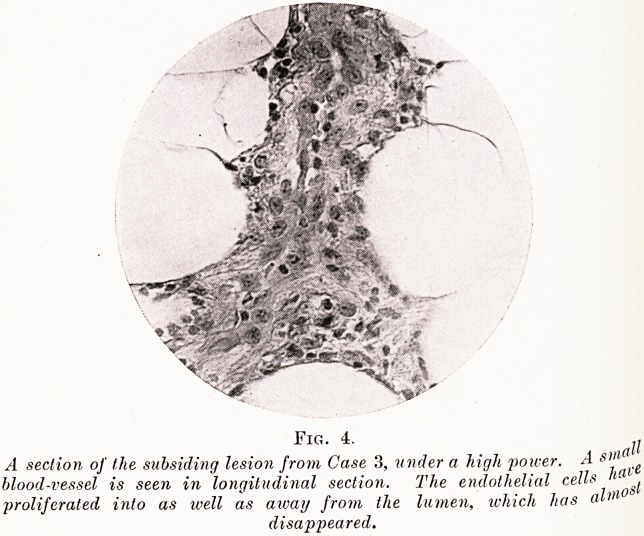# A Note on the Pathology of Erythema Nodosum
*A Martyn Memorial Scholarship Essay.


**Published:** 1928

**Authors:** A. C. Fisher


### A NOTE ON THE PATHOLOGY OF ERYTHEMA NODOSUM.*

BY

A. C. FISHER.

---

THE current views on this subject, which are somewhat conflicting, are briefly set out here.

(*a*) A bacteriæmia due to a variety of organisms or one specific organism. Rosenow[1] found a pleomorphous diphtheroid, Finger[2] a streptococcus, Manzer a staphylococcus. Hadfield and Symes could find no organisms in the nodules they examined.

(*b*) A focal streptococcal infection of the teeth, tonsils or nasal sinuses (Connell[3]).

(*c*) An allergic manifestation of one or more diseases, *e.g.* acute rheumatism and tuberculosis.

(*d*) A manifestation of tuberculosis—"the rash of tuberculosis."

Pusey[4] described the lesion as "septic infarcts of the corium." Rosenow found that the lesion centred round a small artery. Low,[5] however, says that it is round a subcutaneous vein. Poncet and Landouzy[6] claim to have found tubercle bacilli in the lesion, and several authors describe giant cells, from which they infer the condition to be tuberculous.

* A Martyn Memorial Scholarship Essay.

The blood changes are also in dispute.

Hoyer[7] finds an eosinophilia, and concludes that the condition is anaphylactic in origin. Others report variously, a lymphocytosis, neutrophilia and monocytosis.

Four cases were investigated by the present writer. In these the blood picture was examined, and nodules excised and investigated histologically.

**Case 1.**—A boy aged 10, admitted on about the seventh day of the illness. The rash had almost faded. The leucocytic count showed a lymphocytosis of 8,500, while the polymorphonuclears were 5775. There was no eosinophilia.

Serial sections of the skin nodule were cut and stained with iron hæmatoxylin and van Gieson. The cutis was quite unaffected, and the lesion lay in the subcutaneous fatty tissue. An arteriole of moderate size was cut obliquely in the sections, and showed acute inflammatory changes (Fig. 1). Its walls were thickened and the media and adventitia showed loss of structure and cell infiltration ; the tunica intima was fragmented. The lumen did not contain thrombus. Small hemorrhages had occurred into the surrounding tissues where hyperæmia was marked.

Immediately adjacent to the arteriole was a large area of dense cell infiltration, and smaller areas of a similar nature lay scattered in the surrounding fat. The infiltrating cells consisted chiefly of connective tissue and plasma cells with a few lymphocytes and endothelial cells. No polymorphonuclear leucocytes were to be seen.

**Case 2.**—A woman of middle age, admitted while the rash was at its height. She also showed phlyctenules in the eyes, which appeared and disappeared with the rash. The blood showed a marked polynuclear leucocytosis, and no eosinophiles were seen.

The skin lesion was investigated as before. An arteriole lay at the centre of the lesion. It showed the same signs of acute inflammation as in the previous case, but the lumen of the vessel was choked with thrombus (Fig. 2). A vein near by contained a clear clot but showed no signs of inflammation. Many capillaries were occluded and their endothelium was proliferating. Areas of cell infiltration were present; in this case polymorphonuclear cells were in evidence, and there were a few large mononuclears and many endothelial cells.

**Case 3.**—A young woman, admitted while the rash was at its height. A blood count on admission showed a leucocytosis of 15,600:—

| | | |
|---|---|---|
| Polynuclears | .. | 11,900 |
| Lymphocytes | .. | 3,437 |
| Basophiles | .. | 234 |
| Eosinophiles | .. | 0 |

A week later the total white cells were 11,243.

| | | |
|---|---|---|
| Polynuclears | .. | 5,610 |
| Lymphocytes | .. | 4,738 |
| Mononuclears | .. | 350 |
| Basophiles | .. | 85 |
| Eosinophiles | .. | 22 |

Two nodules were excised, one recent and the other subsiding. (*a*) *Recent.*—One medium-sized arteriole lay at the centre of the lesion. It was choked with thrombus and its walls showed acute inflammatory changes (Fig. 3). There was marked hyperæmia. The endothelium of the capillaries and terminal arterioles showed active proliferation. Areas of cell infiltration were not so numerous as in the older lesions. The cells in them included polymorphonuclears, some large mononuclear leucocytes, a few lymphocytes, and endothelial cells.

(*b*) *Subsiding.*—No blood vessel of any size was present in the sections. The terminal arterioles and capillaries showed inflammatory changes with proliferation of their endothelium

(Fig. 4). There were many small areas of cell infiltration in the subcutaneous fat. In these the endothelial cells presented a striking feature. They were much larger than in the recent lesion and frequently contained two nuclei ; the cytoplasm had a foamy appearance and contained granular debris. The polymorphonuclears of the recent lesion have disappeared, but a few lymphocytes are present.

Portions of (*a*) and (*b*) were fixed in alcohol and examined for tubercle bacilli, but none were found. Other sections were stained by Gram's method and other micro-organisms searched for, without result.

**Case 4.**—A boy 11 years of age, admitted with slight pyrexia and a well-marked rash, a fresh exacerbation of which appeared a few days after admission. A series of blood counts was made over a period of twelve days, the results of which are shown in the graph below :—

PLATE II.

FIG. 1.

*A low magnification of the lesion from Case 1, showing that it is situated in the subcutaneous areolar tissue.*

FIG. 2.

*Low power view of nodule from Case 2. Shows an inflamed and thrombosed arteriole beside which there is a venule, which is unaffected by the inflammation but distended with serum*

PLATE III.

Fig. 3.

*A section of the more recent nodule from Case 3, showing inflammatory changes in the wall of an arteriole, the lumen of which has become occluded by leucocytes.*

Fig. 4.

*A section of the subsiding lesion from Case 3, under a high power. A small blood-vessel is seen in longitudinal section. The endothelial cells have proliferated into as well as away from the lumen, which has almost disappeared.*

No eosinophilia was noticed in any of these counts, the highest reached being 1·6 per cent. on the twelfth day. The leucocytic reaction is therefore that of acute infection, with an early neutrophilia and a post-infective lymphocytosis.

The nodule, when examined, showed a lesion similar in all essentials to the previous cases. At the centre was an inflamed arteriole containing thrombus, and around was an area of cell infiltration. The capillaries were also affected. The infiltrating cells consisted of proliferating fibrous tissue cells, polymorphonuclear leucocytes, a few lymphocytes and large mononuclear leucocytes, and some endothelial cells. Sections were stained and examined for tubercle bacilli and other micro-organisms, but none were found.

### SUMMARY.

The local lesion in erythema nodosum is essentially an acute arteriolitis affecting the small vessels under the skin; capillaries as well as arterioles are affected, though the venules do not seem to be involved. Associated with this are areas of cell infiltration in the fat surrounding the affected vessel; these are also of an acute inflammatory nature, and do not resemble granulomata. No giant cells were found, though large endothelial cells were a feature of some of the lesions. No tubercle bacilli or other micro-organisms were observed.

The leucocytic reaction was typical of an acute infection, being an early polynuclear leucocytosis followed by a post-infective lymphocytosis.

I have to thank Dr. J. O. Symes for his kindness in encouraging me to record these observations on patients under his care in the Bristol General Hospital, and Professor Hadfield for his advice and help.

### REFERENCES.

[1] Rosenow, *Journ. of Infectious Diseases*, 1915, vol. xvi., p. 240.
[2] Finger, *B. M. J.*, 1921, ii. 556.
[3] Connell, *Canadian Medical Journal*, 1925, xv. 785.
[4] Pusey, *Principles and Practice of Dermatology.*
[5] Low, *Anaphylaxis and Sensitisation.*
[6] Poncet, Thése de Lyons, 1905–6.
[7] Hoyer, *Acta Medica Scandinavica*, 1923, vol. lvii., p. 587.

## Figures and Tables

**Figure f1:**
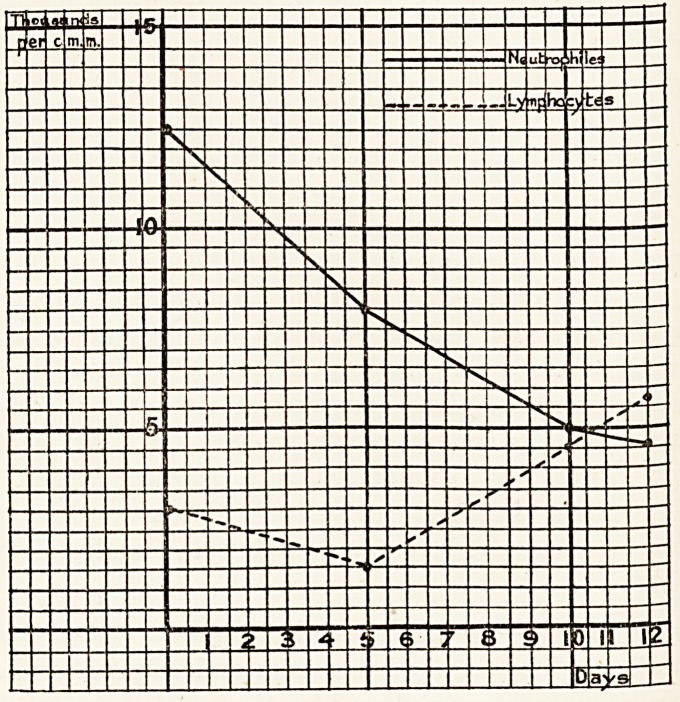


**Fig. 1. f2:**
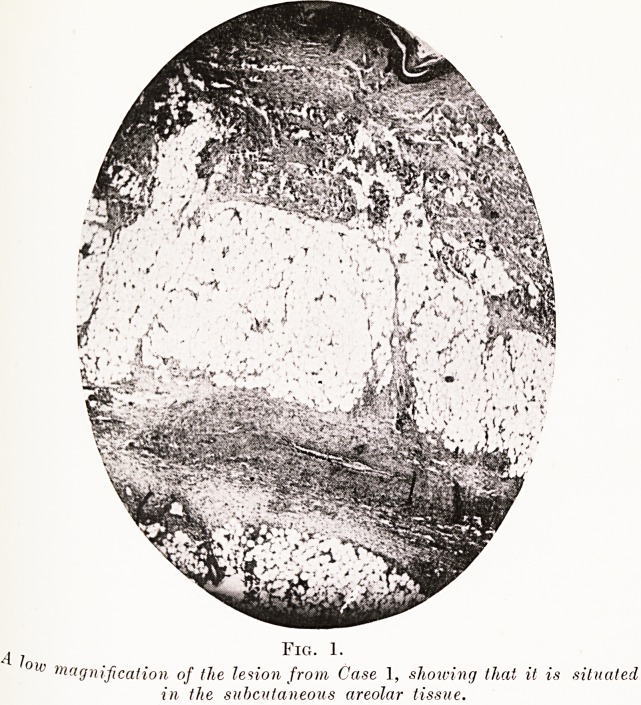


**Fig. 2. f3:**
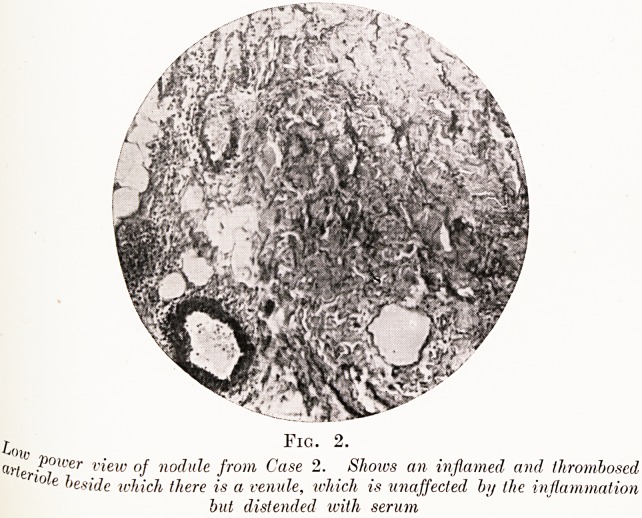


**Fig. 3. f4:**
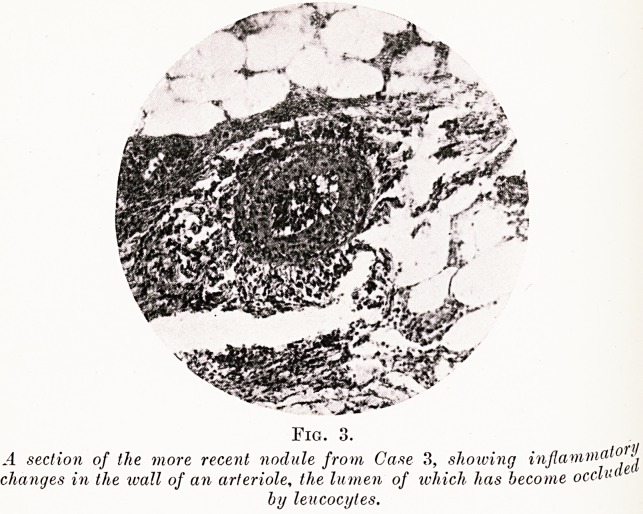


**Fig. 4. f5:**